# The Cardioprotective Effect of Metformin in Doxorubicin-Induced Cardiotoxicity: The Role of Autophagy

**DOI:** 10.3390/molecules23051184

**Published:** 2018-05-15

**Authors:** Rita Zilinyi, Attila Czompa, Andras Czegledi, Andrea Gajtko, Dora Pituk, Istvan Lekli, Arpad Tosaki

**Affiliations:** 1 Department of Pharmacology, Faculty of Pharmacy, University of Debrecen, Nagyerdei Krt 98, 4032 Debrecen, Hungary; zilinyi.rita@pharm.unideb.hu (R.Z.); czompa.attila@pharm.unideb.hu (At.C.); czegledi.andras@pharm.unideb.hu (An.C.); pituk.dora@gmail.com (D.P.); lekli.istvan@pharm.unideb.hu (I.L.); 2 Department of Anatomy, Histology and Embriology, Faculty of Medicine, University of Debrecen, Nagyerdei Krt 98, 4032 Debrecen, Hungary; gajtko.andrea@med.unideb.hu

**Keywords:** doxorubicin, metformin, AMPK, autophagy, cardiotoxicity, heart failure

## Abstract

The molecular mechanisms underlying doxorubicin-induced cardiotoxicity are still being investigated, but are known to involve oxidative stress, mitochondrial dysfunction, and the dysregulation of autophagy. The objective of the current study was to examine the protective role of metformin and its effect on autophagy in doxorubicin-induced cardiotoxicity. Sprague–Dawley rats were divided into four groups at random. The doxorubicin-treated group received doxorubicin (3 mg/kg every second day) intraperitoneally. The metformin-treated group received 250 mg/kg/day metformin via gavage. The doxorubicin + metformin-treated group received both at the above-mentioned doses. The control group received vehicle only. Following the two-week treatment, the hearts were isolated, and cardiac functions were registered. Serum levels of lactate dehydrogenase (LDH), creatine kinase iso-enzyme MB (CK-MB) enzyme, Troponin T, and cardiac malondialdehyde (MDA) were also measured. Heart tissue samples were histopathologically examined by using Masson’s trichrome staining and Western blot analysis was conducted for evaluating the expression level of AMP-activated protein kinase (AMPK) and autophagy-associated proteins beclin-1, LC3B-II, and p62, respectively. The results revealed that treatment with metformin conferred increased cardiac protection against the development of cardiotoxicity manifested by a significant decrease in serum Troponin T and cardiac MDA levels, and remarkable improvement in heart function in connection with histopathological features. Furthermore, by focusing on the contribution of autophagic proteins, it was found that metformin normalised autophagy, which may help cardiomyocytes survive doxorubicin-induced toxicity. These results promote the use of metformin, which would be a preferable drug for patients receiving doxorubicin.

## 1. Introduction

Doxorubicin (DOX) is a highly effective and widely-used non-selective class I anthracycline antibiotic which is frequently incorporated in the treatment of acute leukemia, malignant lymphoma, and several solid tumors [1,2]. However, the efficacy of DOX is hindered due to the cumulative and irreversible cardiotoxicity, which is considered as the most prominent side effect, consequently leading to the development of left ventricular dysfunction, dilated cardiomyopathy, and heart failure years after the treatment has been stopped [3,4,5]. The molecular mechanisms underlying DOX-induced cardiotoxicity are multifactorial and are still unclear, but mitochondrial dysfunction, oxidative stress, apoptosis, and dysregulation of autophagy are involved [6,7]. Furthermore, the heart is very susceptible to DOX-induced lipid peroxidation and toxicity because of its high energy requirement and mitochondrial density [8].

Autophagy is a highly conserved process which is aimed to maintain cell and tissue homeostasis, and involves the elimination of damaged and long-lived organelles under both physiological and pathological conditions [9], including energy and oxygen status, nutrient starvation, and modification in metabolism [10]. Three types of autophagy can be described including microautophagy, chaperone-mediated autophagy, and macroautophagy. The autophagy term hereafter refers to macroautophagy in this study. The role of autophagy in cardiac tissue is apparently dual from the view of survival or death; it depends on the type and the duration of the stress [11]. Several studies have found that DOX treatment affects autophagy, however, it is still not clearly elucidated how DOX alters this process. Previous studies on this matter have shown many controversial results [7,12]. Recently reported studies have demonstrated that DOX induces autophagy; however, it causes dysregulation in the autophagic flux and the autophagic process cannot be completed [13]. These findings are also supported by Hill and co-workers, showing that the administration of DOX inhibits the lysosomal acidification, thus causing disruption in the autophagy flux [13]. In addition, Tokarska et al. reported that DOX can cause dysregulation in most processes of myocardial energy metabolism, such as the AMP-activated protein kinase (AMPK) signaling pathway [14]. AMPK is a major sensor of cell energetic homeostasis. Low cellular energy levels and increased reactive oxygen species (ROS) result in the phosphorylation and activation of AMPK, which is able to induce autophagic processes [15,16,17].

Metformin (MET) is an orally used first-line anti-diabetic drug for the treatment of type 2 diabetes. Several studies reported that application of MET decreases mortality and cardiovascular end-points of type 2 diabetes and has protective effects in cardiac function [18,19].

Recently, several studies have found that MET activates AMPK, and through the AMPK signaling pathway it induces cardiac autophagy and improves cardiac functions. Indeed, Kobashigawa and colleagues demonstrated that cardioprotective effect of MET against DOX-induced toxicity is mediated via upregulation of AMPK and its downstream target molecules [20]. However, high doses of MET treatment induce the same alteration in the AMPK pathway, but its protective effect is lost. The authors suggested that this could be due to the downregulation of platelet-derived growth factor receptor. Furthermore, silencing of adiponectin receptors supressed AMPK activation and cell viability in MET- and DOX-treated cells [21]. 

Nowadays, several extensive studies have investigated the role of autophagy in DOX-induced cardiotoxicity, but we are one of the first research groups to investigate the effect of MET in the autophagic process in DOX-treated animals under in vivo followed by ex vivo conditions.

Various studies have revealed that boosting or restoring autophagy could help the cardiomyocytes to survive during DOX therapy. In the present study, we co-administered DOX and MET in order to investigate the role of MET in the autophagic process and its cardioprotective properties in DOX-induced cardiotoxicity. Thus, our investigation may offer further understanding of the role of cardiac autophagy in DOX-treated animal subjects. In addition, our hypothesis was that MET could activate AMPK, restore autophagy, and improve cardiac function, which may consequently mean that DOX co-administered with MET help the cardiomyocytes to survive. This could be a promising new strategy for patients suffering from cancer and receiving the DOX regimen. Moreover, it remains a challenge to find an effective agent which might be combined with DOX that is able to reduce its cardiotoxicity, whilst maintaining its efficacy and safety in tumor therapies.

## 2. Results

### 2.1. DOX and MET Effects on Cardiac Function in Isolated Hearts

**Figure 1:**
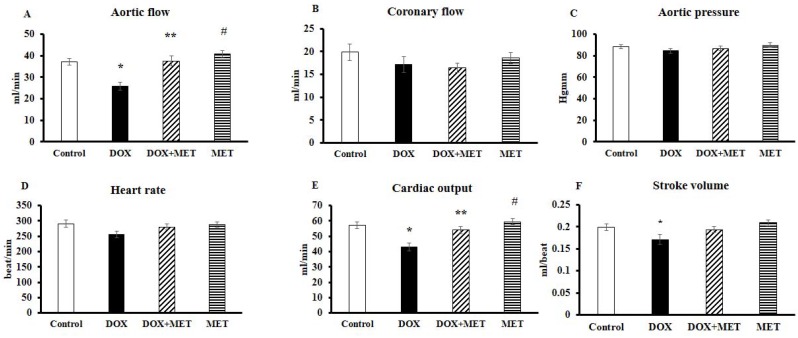
Effect of doxorubicin (DOX) and metformin (MET) on cardiac function (*n* = 17–19) of isolated “working hearts” from rats after the 2-week treatment. Cardiac functions including aortic flow (**A**), coronary flow (**B**), aortic pressure (**C**), heart rate (**D**), cardiac output (**E**), and stroke volume (**F**) were recorded after 10 min in “working mode”. Results are provided as average magnitude of each cardiac value within a group of animals ± standard error of mean (SEM). The significance of differences among groups were evaluated with one-way analysis of variance (ANOVA) followed by the Tukey comparison test. *p* values of 0.05 or less were considered significant in each graph; * Significant difference the control group vs. DOX group; ** Significant difference DOX group vs. DOX+MET group; ^#^ Significant difference DOX group vs. MET group.

Figure 1 shows the cardiac function in hearts isolated from the animals 24 h after the last dose of MET and/or DOX. Graph A demonstrates the aortic flow (AF), which was significantly lower (*p* < 0.05) in the DOX group compared to the control group. This value was significantly higher in DOX+MET group. Graph B expresses the coronary flow (CF), which showed no significant differences between the control and the treated groups. Graph C displays no significant difference in aortic pressure (AOP) among the four studied groups. We observed lower heart rate (HR) (not significant) in DOX-treated group compared to the drug-free control group, while in the DOX+MET group, the values were almost at the same level as the control group (Figure 1D). Furthermore, as per Figure 1E,F, a significant decrease (*p* < 0.05) in cardiac output (CO) and stroke volume (SV) in DOX group compared to the control group was detected. In the DOX+MET treated group, the CO was at a significantly higher (*p* < 0.05) level, while SV was slightly increased (not significant).

### 2.2. DOX and MET Effects on Serum Biomarkers

**Figure 2:**
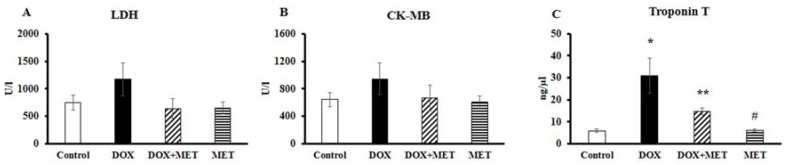
Effect of doxorubicin and metformin on serum biomarkers (*n* = 10–12) after the 2-week treatment. lactate dehydrogenase (LDH) (**A**), creatine kinase iso-enzyme MB (CK-MB) isotype (**B**) and Troponin T (**C**) levels were obtained from blood samples taken from the left jugular vein. Results are provided as average magnitude of each value within a group of animals ± SEM. The significance of differences among groups was evaluated with one-way analysis of variance (ANOVA) followed by the Tukey comparison test. *p* values of 0.05 or less were considered significant in each graph; * Significant difference the control group vs. DOX group; ** Significant difference DOX group vs. DOX+MET group; ^#^ Significant difference DOX group vs. MET group.

Figure 2 depicts that administration of 6 × 3 mg/kg of DOX alone resulted in a slight increase in serum enzyme activities, such as lactate dehydrogenase (LDH) (Figure 2A) and creatine kinase iso-enzyme MB (CK-MB) isotype (Figure 2B) compared to the control group, meanwhile in the DOX+MET group, these enzymes remained at a lower level compared to the DOX-treated group. In addition, we have found a significantly higher (*p* < 0.05) Troponin T level in DOX group compared to the control (Figure 2C), while 250 mg/kg MET treatment resulted in a significantly lower level of Troponin T compared to the DOX group, indicating that MET is able to reduce the detrimental effect of DOX.

### 2.3. DOX and MET Effects on Lipidperoxidation

**Figure 3:**
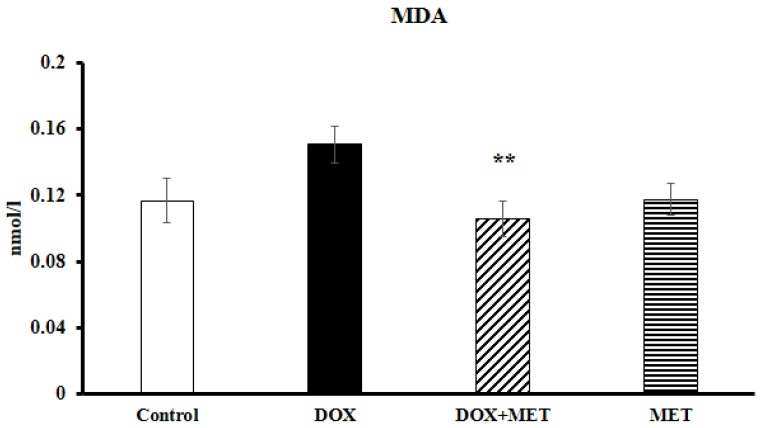
Effect of doxorubicin and metformin on the degree of lipid peroxidation (*n* = 10–12) after the 2-week treatment. Malondialdehyde level was measured from cardiac tissues by using a lipid peroxidation assay. Results are provided as average magnitude of each value within a group of animals ± SEM. The significance of differences among groups were evaluated with one-way analysis of variance (ANOVA) followed by the Tukey comparison test. *p* values of 0.05 or less were considered significant in each graph; ** Significant difference DOX group vs. DOX+MET group; MDA: malondialdehyde.

One of the major contributors to DOX toxicity is oxidative stress. The results of the present study showed that injection of 6 × 3 mg/kg DOX was associated with a considerably elevated level of malondialdehyde (MDA) compared to the control group (Figure 3). While in the DOX+MET treated group, the MDA level was significantly lower (*p* < 0.05) than in the DOX group.

### 2.4. DOX and MET Effects on Histopathological Features

**Figure 4:**
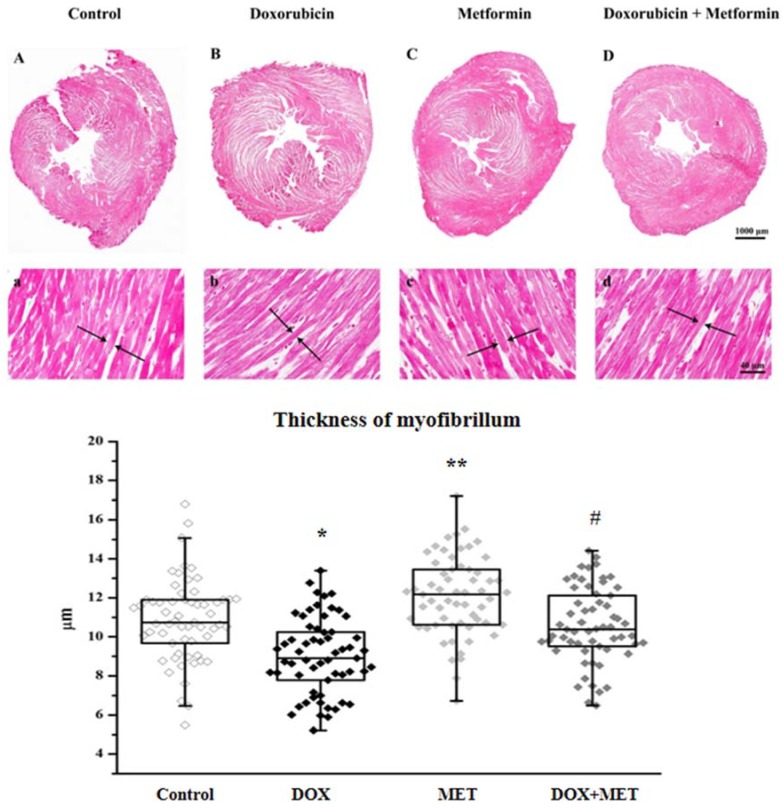
Effect of doxorubicin and metformin on myocardial tissue (*n* = 3 different slides/group and 2 different fields in each slide and 10 myocytes/field) after the 2-week treatment. Representative photomicrographs of rat heart cross sections of control (**A**,**a**), DOX (**B**,**b**), MET (**C**,**c**), DOX+MET (**D**,**d**) magnification 4× and 40×. The thickness of myofibrillum is seen in the graph below the corresponding picture. The sections were studied under an Olympus CX-31 microscope and photomicrographs were taken using Olympus DP74 camera (Olympus Corporation, Tokyo, Japan) at 4× (scale bar 1000 μm) and 40× (scale bar 40 μm) magnification. Results are provided as average magnitude of each value within a group of animals ± SEM. The significance of differences among groups was evaluated with Mann–Whitney test. *p* values of 0.05 or less were considered significant in each graph; * Significant difference the control group vs. DOX group; ** Significant difference DOX group vs. DOX+MET group; ^#^ Significant difference DOX group vs. MET group.

DOX-induced cardiotoxicity was further assessed using trichrome staining. Representative cross sections of each group and the graph displaying myofibrillar thickness are shown (Figure 4). Cardiac tissues were stained with aniline blue for detection of fibrillar collagen and hence fibrosis. Two weeks of treatment with DOX and/or MET did not show any visible differences in the amount of collagen deposit in the treated groups. However, it is important to highlight that the myocytes in DOX-treated groups were significantly thinner (*p* < 0.05) than in the control group. Furthermore, the diameter of myocytes in the DOX+MET group was almost the same as the control group.

### 2.5. DOX and MET Effects on AMPK and Autophagic Markers

**Figure 5:**
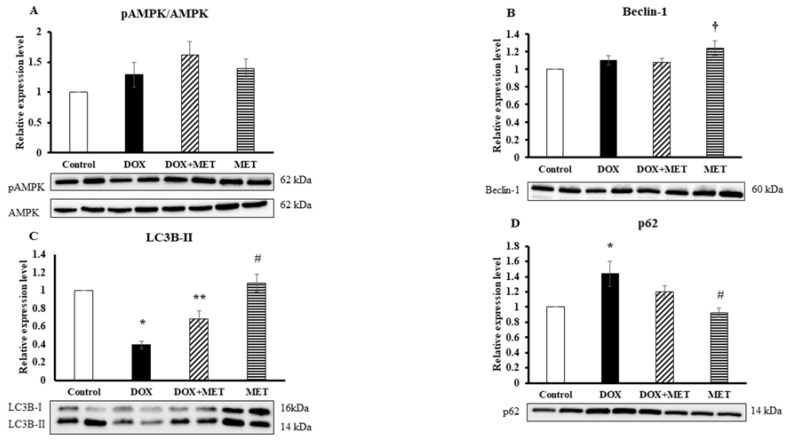
The effect of doxorubicin and metformin on the expression level (*n* = 7–8) of pAMPK/AMPK (**A**), Beclin-1 (**B**), LC3B-II (**C**), and p62 (**D**) after 2-week treatments. The expression level of proteins in left ventricular tissues was evaluated using Western blot analysis. Results are provided as average magnitude of each value within a group of animals ± SEM. The significance of differences among groups was evaluated with one-way analysis of variance (ANOVA) followed by the Tukey comparison test. *p* values of 0.05 or less were considered significant in each graph; * Significant difference the control group vs. DOX group; ** Significant difference DOX group vs. DOX+MET group; ^#^ Significant difference DOX group vs. MET group; ^†^ Significant difference the control group vs. MET group.

Left ventricular tissue levels of AMPK were elevated in all three groups in comparison with the control value (Figure 5A), but these changes were not at a significant level. Beclin-1 showed a significantly increased (*p* < 0.05) expression level in the MET group compared to the control group, while the DOX and DOX+MET groups did not show any changes in comparison with the control group (Figure 5B). LC3-II was significantly decreased (*p* < 0.05) in DOX group compared to the control group, while in the DOX+MET group its expression was increased compared to the DOX group (Figure 5C). For p62, a protein that recognizes toxic cellular waste, which is consequently scavenged by a sequestration process, we observed the exact opposite change. A significantly increased (*p* < 0.05) p62 level was detected in the DOX group compared to the control group, meanwhile, for DOX co-administered with MET (DOX+MET), the expression of p62 was considerably lower (Figure 5D), indicating that DOX treatment impaired autophagic protein clearance and it is restored via MET treatment.

## 3. Discussion

The major limitation of DOX treatment is the acute or chronic cardiac toxicity, which may result in heart failure [22]. Acute toxicity could be reversible with adequate treatment; however, the life expectancy of patients possessing DOX-induced heart failure is currently unclear. The underlying mechanism appears to be multifactorial; however, enhanced oxidative stress is one of the major contributors [23]. Indeed, cells fail to cope with an enhanced amount of reactive oxygen and nitrogen species and enhanced oxidative stress leads to DNA and protein damage and mitochondrial dysfunction [24]. In line with the literature, our results also show elevated oxidative stress evidenced by an enhanced level of MDA in DOX-treated animals. Increased oxidative stress was accompanied by impaired left ventricular functions including AF, CO, and SV in DOX-treated animals compared to the control group. MET-only treatment did not significantly alter heart function or MDA levels. However, our results revealed that MET co-treatment decreased oxidative stress and improved myocardial function. In the present study, cardiac damage induced by DOX and serum levels of LDH, CK-MB, and Troponin T were measured. Elevations in LDH and CK-MB levels represent their leakage from the damaged membranes of cardiomyocytes into circulation and were previously shown to be indicators of cardiotoxicity [25,26]. The administration of 6 × 3 mg/kg DOX alone resulted in a slight elevation in serum enzyme levels in LDH and CK-MB, and serum Troponin T levels, which refer to the development of cardiotoxicity of DOX. Troponin T molecules consisting of amino acid sequences that are found only in cardiac tissue, making it highly specific for detecting cardiac damage. Meanwhile, in the DOX+MET group, the levels of LDH and CK-MB remained at a lower level, and a significantly lower level of Troponin T was also detected. Our results revealed that the daily administration of MET significantly attenuated the rise of LDH, CK-MB, and Troponin T enzyme levels during the DOX treatment, while the administration of MET alone did not show any significant changes in serum enzyme levels. Confirming the cardioprotective effect of MET in DOX-induced cardiac toxicity, histopathological analysis revealed that DOX treatment altered the size of myocytes, which was restored in the presence of MET treatment. Earlier, Ashour and co-workers reported myofibrillar loss and derangement with abnormal mitochondria in DOX-treated myocardium, which was partially normalized when MET was administered to animals [27]. Furthermore, MET treatment decreased the level of the apoptotic caspase 3 and enhanced the level of antiapoptotic Bcl-2 proteins, suggesting that MET possesses antiapoptotic properties in this model [28]. Interestingly, sitagliptin, another antihyperglicaemic agent, showed more potent cardioprotective effects.

Considering autophagy as a mechanism to restore energy status and to clear damaged macromolecules [29], the expression of LC3B-II was studied, which is considered as a major marker of macroautophagy. Upon DOX treatment, a significantly reduced level of LC3B-II protein expression was found, suggesting that suppression of autophagy contributes to the detrimental effect of DOX. Co-treatment with MET significantly enhanced the level of LC3B-II in comparison with DOX treatment, suggesting that MET treatment normalized the autophagic process. Results with p62 protein strongly support this hypothesis. One function of p62 is directing ubiquitinated protein to the autophagosome for degradation. An enhanced level of p62 indicates impaired autophagic flux [30], and an enhanced level of p62 in DOX-treated group suggests that autophagy is malfunctioning.

The alterations of these proteins were not significant between the control and DOX+MET treated groups. Based on these two observations, we may assume that MET treatment normalized the autophagic degradation. However, based on these results, we cannot conclude that MET itself normalized the autophagic processes that leads to cardioprotection, or the cardioprotection induced by MET leads to normalization of autophagic processes. Our results support the suggestion that induction of autophagy before and during DOX treatment serves as a protective mechanisms against cardiovascular complications [10].

In order to study the underlying mechanisms of autophagy induction, the phosphorylation level of AMPK was studied in different groups. AMPK is an energy sensor of the cells, and MET induces a mild inhibition of respiratory-chain complex I in the mitochondria, thereby influencing the AMP/ATP ratio, leading to AMPK activation [31]. As expected, an enhanced level of p-AMPK in MET group was detected, since AMPK is one of the target molecules of MET medication. The literature is more complex regarding the effect of DOX treatment on the mechanism of AMPK activation/deactivation. The same level of p-AMPK, like in samples originated from DOX-treated group, was observed, however, it was not accompanied by enhanced autophagy. It is speculated that the enhanced phosphorylation of AMPK is not a sufficient trigger to induce functioning autophagy if DOX is present, or even if autophagy is being initiated, the whole process is not terminated. Moreover, an elevated level of p-AMPK in animals treated with DOX and MET at the same time was measured, which was accompanied by a decreased level of p62, indicating that autophagy is completed and damaged macromolecules are cleared.

Taken together, the results presented herein confirmed the cardioprotective ability of MET in DOX-induced cardiac complications. The obtained results suggest that DOX treatment impairs the autophagic processes, and damaged macromolecules cannot be degraded in the cells, and co-administration of MET with DOX normalized the autophagic activity and confers cardioprotection. However, to draw a definitive cause–effect relationship between autophagy and MET treatment, the autophagic flux needs to be directly examined.

## 4. Materials and Methods

### 4.1. Animals

The experiments were accomplished using adult female rats (Charles River Laboratories), with a body weight range of 250–300 g. All animals were housed and treated according to the “Principles of Laboratory Animal Care” formulated by the National Society for Medical Research and the “Guide for the Care and Use of Laboratory Animals” prepared by the National Academy of Sciences and published by the National Institutes of Health (NIH Publication no. 86-23, revised in 1996). Maintenance and treatment of animals used in this study was additionally approved by the Institutional Animal Care and Use Committee of the University of Debrecen, Debrecen, Hungary (project ID: 3/2012/DE MAB, 03/2012–03/2017). The animals were housed in wire-bottomed cages throughout the study and were maintained on 12:12-h light-dark cycle; and provided with laboratory rodent chew pellets and water ad libitum.

### 4.2. Experimental Design and Treatment Protocol

Sprague–Dawley rats were randomly divided into four groups as follows I., control group—animals received water per os, and saline intraperitoneally for a time period of 2 weeks; II., MET group—animals received MET at a dose of 250 mg/kg every day orally via gavage [32], and were injected with saline intraperitoneally; III., DOX group—animals received DOX at the dose of 3 mg/kg every second day intraperitoneally [27] (the cumulative dose was 18 mg/kg), and received water orally; IV., DOX+MET group—animals received DOX intraperitoneally every second day and MET each day orally at the above-mentioned dose. MET was dissolved in saline, and animals were sacrificed 24 h after the last dose of MET and DOX. A schematic representation of the treatment protocols and methods can be found in Supplementary Materials.

### 4.3. Isolated Working Heart Preparation and Cardiac Function Assessment

Following the 2-week treatment with vehicle, MET, and DOX respectively rats were anesthetized with i.p. injection of pentobarbital (60 mg/kg), and heparin (1000 IU/kg) was intraperitoneally administered as an anticoagulant. Blood samples were collected from the left jugular vein. Then, thoracotomy was carried out under terminal anesthesia, followed by excision of the heart. The aorta was cannulated and perfused in Langendorff “non-working” mode. Oxygenated Krebs–Henseleit bicarbonate buffer (118.5 NaCl, 4.7 KCl, 2.5 CaCl_2_ × 2H_2_O, 25 NaHCO_3,_ 1.2 KH_2_PO_4_, 1.2 MgSO_4_, and 10.0 glucose (in mM) was the perfusion medium. In the meantime, the pulmonary vein was also cannulated, and the system was switched to the ”working” mode, as previously described by Neely et al. [33], modified by Yamamoto et al. [34] and Tosaki and Hellegouarch [35]. After 10 min of aerobic perfusion, the basic cardiac function was registered. Thus, aortic pressure (AOP) and heart rate (HR) were registered using a computer acquisition system (ADInstruments, PowerLab, Castle Hill, Australia). Coronary flow (CF) was obtained by timed collection of the coronary flow, and aortic flow (AF) was measured by a calibrated flow-meter. Cardiac output (CO) was calculated as the sum of AF and CF, and stroke volume (SV) was generated as the quotient of CO/HR.

### 4.4. Assessment of Serum Biomarkers

Blood samples were obtained from the left jugular vein, and the serum was separated for measurement of LDH, CK-MB and Troponin T (TrT). For the in vitro quantitative determination of the serum biomarkers electrochemiluminescence immunoassay “ECLIA” was used and measured by Roche/Hitachi cobas immunoassay analyzers.

### 4.5. Histopathological Examination

For histopathological examination, cardiac tissues were collected from all studied groups and dissected and fixed in 4% formaldehyde solution. The heart samples were embedded in paraffin, and five micron-thick sections were sliced. Standard histological methods (xylol) were used in order to remove paraffin, and the samples were passed through a gradual alcohol series and hydrated. Trichrome staining was used to demonstrate the general histological structure; it is a three-color staining process, which differentially stains the nucleus, muscle tissue, and collagen. It was performed by using Trichrome Stain Kit (Abcam, Cambridge, UK). The sections were studied under Olympus CX-31 microscope and photomicrographs were taken using Olympus DP74 camera (Olympus Corporation, Tokyo, Japan) at 4× and 40× magnification. The remaining portion of the heart was stored at −80 °C for lipid peroxidation assay and Western blot analysis.

### 4.6. Lipidperoxidation Assay

The level of MDA, in the heart tissue, was detected by using a lipid peroxidation (malondialdehyde; MDA) assay kit (Sigma-Aldrich, St. Louis, MO, USA). Lipid peroxidation was determined by the reaction of MDA with thiobarbituric acid (TBA) to form a colorimetric product, proportional to the MDA present. The intensity of the color was measured spectrophotometrically at 532 nm.

### 4.7. Western Blot Analysis

The expression level of AMPK, p-AMPK, Beclin-1, LC3-II, and p62 proteins in left ventricular tissue was evaluated using Western blot analysis. Approximately 300 mg of heart tissues were homogenized by using a polytron homogenizer in isolating buffer (25 mM Tris-HCL, 25 mM NaCl, 1 mM orthovanadate, 10 mM NaF, 10 mM pyrophosphate, 10 mM okadaic acid, 0.5 mM EDTA, 1 mM PMSF, and 1× protease inhibitor cocktail) and centrifuged at 2000 rpm at 4 °C for 10 min. The supernatant was transferred to a new tube and centrifuged at 10,000 rpm at 4 °C for 20 min, and then the supernatant was used as cytosolic fraction. Protein concentration was measured by a BCA Protein Assay Kit (Thermo Scientific, Rockford, IL, USA). A total of 75 μg of protein in each sample was loaded and separated using electrophoresis on a polyacrylamide gel (TGX Stain-Free^TM^ FastCast^TM^ Acrylamide Kit, 12%, Bio-Rad, Hercules, CA, USA) and transferred onto a PVDF membrane. Nonspecific binding sites were blocked with 7% skimmed milk in Tris-buffered saline solution with 0.5% Tween 20 for 1 hour at room temperature. Membranes were incubated overnight at 4 °C with primary antibodies directed against AMPK, p-AMPK, Beclin-1, LC3-II, p62 (Cell Signaling Technology, MA, USA). After washing the membranes, they were incubated with corresponding horseradish-peroxidase-conjugated secondary antibodies for 1.5 h at room temperature and signal intensities for each protein band was detected using Clarity Western ECL Substrate (Bio-Rad, California, USA). The optical density of bands was measured using the ChemiDoc Touch Imaging System (Bio-Rad, California USA). The level of the protein of interest was normalized against the total amount of protein in each lane with the Bio-Rad Image Lab 5.2.1 software (Bio-Rad Laboratories, Inc., Hercules, CA, USA).

### 4.8. Statistical Analysis

The data was analyzed by IBM SPSS Statistics 22.0 statistical software (IBM Corporation, New York, NY, USA) The data are expressed as mean ± standard error of the mean (mean ± 1 SE). The significance of differences among groups was evaluated with one-way analysis of variance (ANOVA) followed by the Tukey comparison test. For the histopathological examination Mann–Whitney test was used to evaluate the data. *p* values of 0.05 or less were considered to be significant.

## 5. Limitation of the Study

One limitation of this study was that the cardiac function and serum biomarkers were measured and examined only 24 h after the administration of the last dose of DOX. Therefore, we were unable to determine the long-term effects of the co-administration of these two drugs. Another limitation of the study is that DOX was intraperitoneally administered, whereas the protocols for patients involve intravenous administration.

## Supplementary Materials

Supplementary Materials can be found online.
