# Supplementary figures and images for: The Cardioprotective Effect of Metformin in Doxorubicin-Induced Cardiotoxicity: The Role of Autophagy
Source: Molecules. 2018 May 15;23(5):1184. doi: 10.3390/molecules23051184 (PMC6100061; doi:10.3390/molecules23051184)

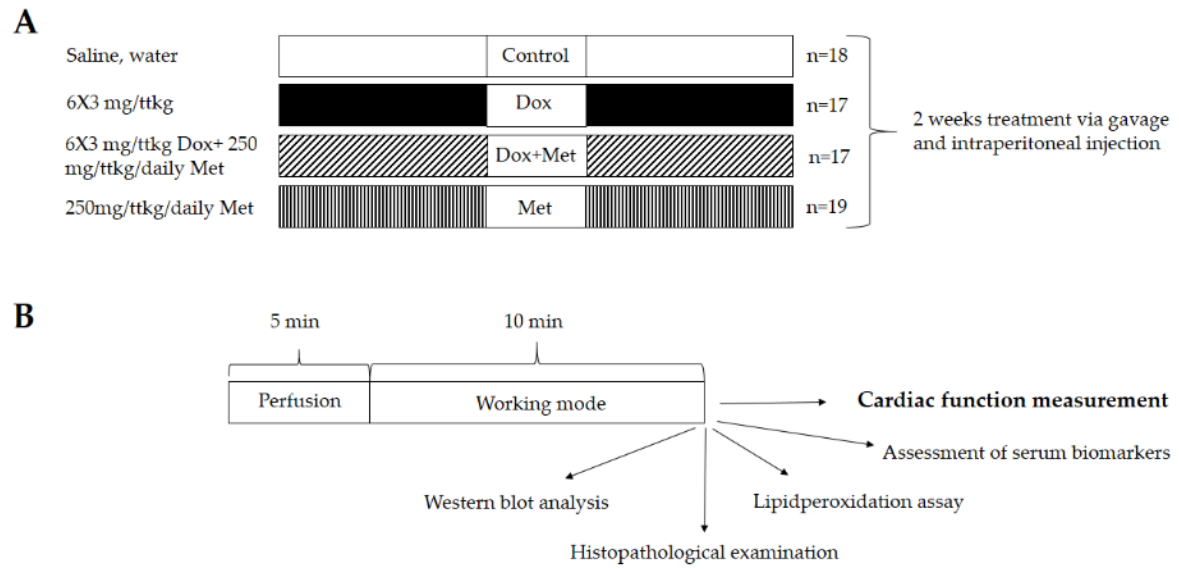

**Figure S1.** Schematic representation of treatment protocols (A) and methods (B).

Supplement: Supplementary file 1 [file molecules-23-01184-s001.pdf]
